# Target sequencing and CRISPR/Cas editing reveal simultaneous loss of *UTX* and *UTY* in urothelial bladder cancer

**DOI:** 10.18632/oncotarget.11207

**Published:** 2016-08-11

**Authors:** Jinwoo Ahn, Kwang Hyun Kim, Sanghui Park, Young-Ho Ahn, Ha Young Kim, Hana Yoon, Ji Hyun Lee, Duhee Bang, Dong Hyeon Lee

**Affiliations:** ^1^ Department of Chemistry, Yonsei University, Seoul, Korea; ^2^ Department of Urology, Ewha Womans University School of Medicine, Seoul, Korea; ^3^ Department of Pathology, Ewha Womans University School of Medicine, Seoul, Korea; ^4^ Department of Molecular Medicine, Ewha Womans University School of Medicine, Seoul, Korea; ^5^ Department of Clinical Pharmacology and Therapeutics, College of Medicine, Kyung Hee University, Seoul, Korea

**Keywords:** urinary bladder neoplasm, chromatin remodeling, UTX, UTY, epigenesis

## Abstract

*UTX* is a histone demethylase gene located on the X chromosome and is a frequently mutated gene in urothelial bladder cancer (UBC). *UTY* is a paralog of *UTX* located on the Y chromosome. We performed target capture sequencing on 128 genes in 40 non-metastatic UBC patients. *UTX* was the most frequently mutated gene (30%, 12/40). Of the genetic alterations identified, 75% were truncating mutations. *UTY* copy number loss was detected in 8 male patients (22.8%, 8/35). Of the 9 male patients with *UTX* mutations, 6 also had copy number loss (66.7%). To evaluate the functional roles of *UTX* and *UTY* in tumor progression, we designed *UTX* and *UTY* single knockout and *UTX*-*UTY* double knockout experiments using a CRISPR/Cas9 lentiviral system, and compared the proliferative capacities of two UBC cell lines in vitro. Single *UTX* or *UTY* knockout increased cell proliferation as compared to *UTX*-*UTY* wild-type cells. *UTX*-*UTY* double knockout cells exhibited greater proliferation than single knockout cells. These findings suggest both *UTX* and *UTY* function as dose-dependent suppressors of UBC development. While *UTX* escapes X chromosome inactivation in females, *UTY* may function as a male homologue of *UTX*, which could compensate for dosage imbalances.

## INTRODUCTION

Several studies have described recurrent somatic mutations and copy number alterations in urothelial bladder cancer (UBC) [1–3]. UBC is characterized by a large number of genetic alterations. It has the third highest mutation frequency after melanoma and lung cancer [4]. Along with well-known bladder cancer-associated genes such as *TP53*, *RB1*, *FGFR*, *PIK3CA*, *HRAS*, *KRAS*, and *TSC1* [5], frequent genetic alterations in genes that regulate processes such as chromatic remodeling as well as sister chromatid cohesion and segregation have been identified [1, 6].

Genetic alterations in chromatin-modifying genes have been reported in multiple types of cancer [7–9]. Intriguingly, chromatin-modifying genes were more frequently mutated in UBC than in any other cancer, and more than 70% of patients with UBC were shown to have genetic alterations in chromatin-remodeling genes [3]. Integrated genomic analysis demonstrated that chromatin-modifying genes comprise the main pathway implicated in UBC. Thus, chromatin modification/remodeling pathways may be promising targets for the treatment of UBC [3].

The Y chromosome has the capacity to regulate target gene expression. Along with its role in sex determination, X-Y paired genes are necessary for normal physiological function [10]. *UTX*, a histone demethylase located on the X chromosome also known as *KDM6A*, is frequently mutated in various types of cancer [11]. Although *UTX* is one of the most frequently mutated genes in UBC, genetic alterations in *UTY*, a paralog of *UTX*, have not been characterized [1–3]. In this study, we performed a genomic analysis of UBC using target capture sequencing. We analyzed somatic mutations and copy number variations in genes involved in chromatin modification. Genetic alterations in two chromatin-modifying genes, *UTX* and *UTY*, were evaluated using knock-out assays and the CRISPR/Cas9 lentiviral system.

## RESULTS

### Target capture sequencing

**Figure 1:**
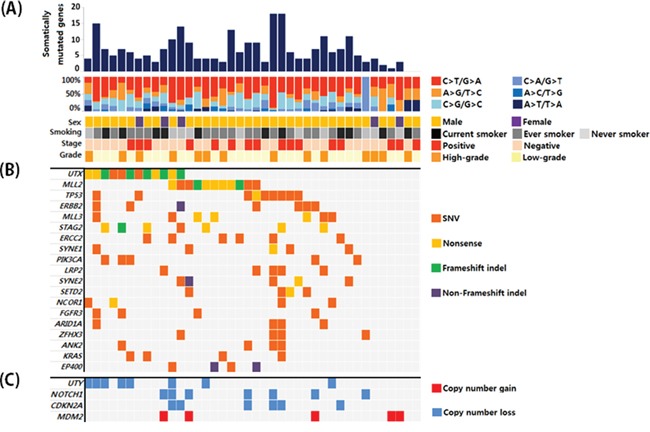
**A.** Overall mutation profiles and patient characteristics. **B.** Genes with somatic mutations that had frequencies ≥ 10% and the corresponding mutation types. **C.** Genes with copy number variations that were detected at a frequency ≥ 10%.

Of a total of 40 UBC samples, 16 were muscle-invasive UBC and 26 were high-grade. The clinical and pathological characteristics of the patients are shown in Supplementary Table S1. All patients underwent surgery for UBC between July 2013 and June 2014. Probes were designed to capture exons in target genes (Supplementary Table S2). We performed target capture sequencing on UBC samples. The mean depth was 425X and 515X for paired tumor and blood samples, respectively. The coverage in the target region was 95.3% and 95.7%, respectively, with an average of ≥ 50 non-duplicated reads (Supplementary Table S3). After somatic mutation calling, synonymous and off-target mutations were rejected. We identified non-synonymous mutations including 157 missense, 36 nonsense, 18 frameshift indels, and 5 non-frameshift indels after common variant removal based on the 1000 Genomes Project (frequency ≥ 1%) (Supplementary Table S4). Overall, 36 of 40 patients (90%) had putative non-synonymous mutations in the target genes. By grouping somatic single nucleotide variants (SNVs) according to the nature of the nucleotide change, we observed enrichment of C:G → T:A transition mutations (45% on average), followed by C:G → G:C transversion mutations (21% on average). These results were consistent with data from a previous bladder cancer study (Figure 1A) [4]. Non-synonymous mutations in 40 bladder cancer samples that had frequencies ≥ 10% are shown in Figure 1B. In addition to somatic mutations, we also searched for somatic copy number alterations. Overall, 27 of 40 patients had somatic copy number alterations in 22 genes. Copy number gains were observed for 9 genes and copy number losses were observed for 13 genes. The genes with copy number alterations with frequencies ≥ 10% are shown in Figure 1C. The total somatic alteration profile is shown in Supplementary Figure S1.

### Genetic alterations in chromatin remodeling genes

**Figure 2:**
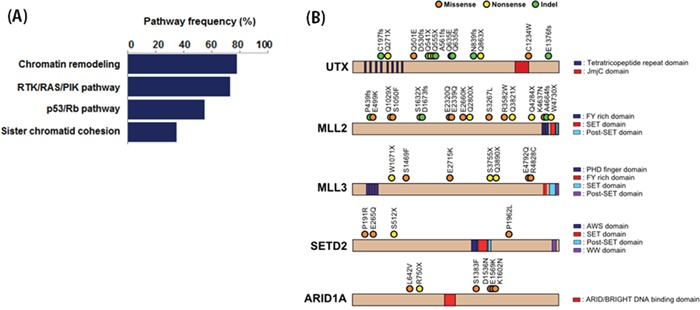
**A.** Pathway enrichment analysis of mutated genes. **B.** The locations of somatic mutations in chromatin remodeling genes that had frequencies ≥ 10%.

The most frequently mutated genes in our cohort were those involved in chromatin remodeling (Figure 2A). Somatic mutations were observed in *UTX*, *MLL2*, *MLL3*, *SETD2*, *ARID1A*, *ARID1B*, *SMARCA4*, *SMARCA2*, *NSD1*, *EP300*, *KDM5B*, *KDM5A*, *CHD6*, and *CREBBP.* Copy number losses were observed in *UTY*, *NSD1*, *SMARCA2*, and *SMARCC2*. Of the 40 patients, 31 (75%) had genetic alterations in chromatin remodeling genes. Additionally, 8 of 21 chromatin remodeling genes had genomic alterations in ≥ 10% of the sample in our analysis cohort (Figure 2B). Chromatin remodeling genes also had a relatively high proportion of truncating mutations compared to other genes in the pathways. Truncating mutations were highly enriched in the two most frequently mutated genes (UTX and MLL2), with frequencies of 75% and 63.6%, respectively.

### Alterations in *UTX* and *UTY* are associated with bladder cancer in men

*UTX*, a histone demethylase located on the X chromosome, was the most frequently mutated gene in our set. Of the 12 patients with *UTX* mutations, 9 (75%) had truncating mutations (4 nonsense and 5 frameshift indels). Interestingly, another chromatin remodeling gene, *UTY*, exhibited the most frequent copy number losses in our study. *UTY* copy number losses were observed in 8/35 male patients (22.8%). *UTY* is a paralog of *UTX.* It is located on the Y chromosome and has 88% amino acid similarity with *UTX. UTX* is subject to X inactivation. While monoallelic loss of *UTX* may be compensated by another copy of the *UTX* gene in females, the allelic role of *UTY* for *UTX* is not clear. Of the 9 men in our dataset with *UTX* mutations, 6 (66.7%) also had *UTY* copy number loss. Simultaneous loss of *UTX* and *UTY* in UBC suggests that *UTY* may be the male homologue of *UTX*.

### Double knock-out of *UTX* and *UTY* in UBC cell lines in vitro

**Figure 3:**
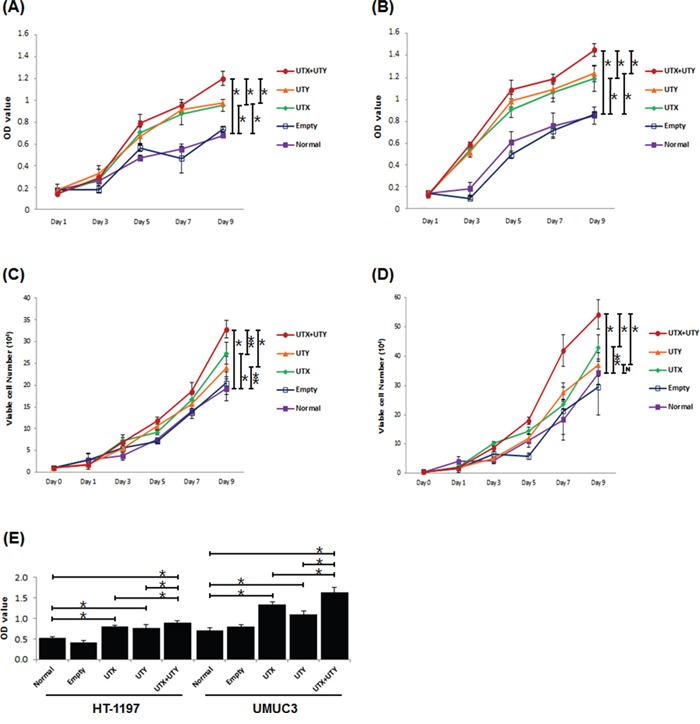
Changes in proliferation between *UTX*/*UTY* single knock-out and *UTX*-*UTY* double knock-out cells (*, P < 0.01; **, P < 0.05; N, not significant) **A.** MTT assays with the HT-1197 cell line. **B.** MTT assays with the UMUC3 cell line. **C.** Cell counting assays with the HT-1197 cell line. **D.** Cell counting assays with the UMUC3 cell line. **E.** BrdU incorporation assays with the HT-1197 and UMUC3 cell lines.

To investigate the role of genomic loss of *UTX* and *UTY* in UBC, we generated *UTX* single knock-out, *UTY* single knock-out, and *UTX*-*UTY* double knock-out UBC cell lines using the CRISPR/Cas9 lentiviral system, and compared cell proliferation in vitro. Cas9 knock-out experiments were based on viral transduction according to the GeCKO protocol [12]. Exon 3 of *UTX* and exon 1 of *UTY* were selected as single guide RNAs (sgRNAs) based on the sgRNA off-target score. Human HT-1197 and UMUC3 cells were infected with *UTX-* and *UTY*-targeting lentiviruses. Untreated or empty-vector lentivirus-treated cells were cultured as controls. Proliferation was assessed in *UTX* single knock-out, *UTY* single knock-out, and *UTX*-*UTY* double knock-out cells and compared to controls using MTT, cell counting, and BrdU incorporation assays. Single knock-out of either *UTX* or *UTY* resulted in enhanced cell proliferation compared to *UTX*-*UTY* wild-type cells. A similar increase in proliferation was observed in *UTX*-*UTY* double knock-out cells compared to single knock-out cells in both the HT-1197 and UMUC3 cell lines (Figure 3A). These results indicate that both *UTX* and *UTY* function as tumor suppressors in UBC, and that loss of *UTY* contributes to UBC progression.

### Gene expression analysis of *UTY* knock-out cells

**Figure 4:**
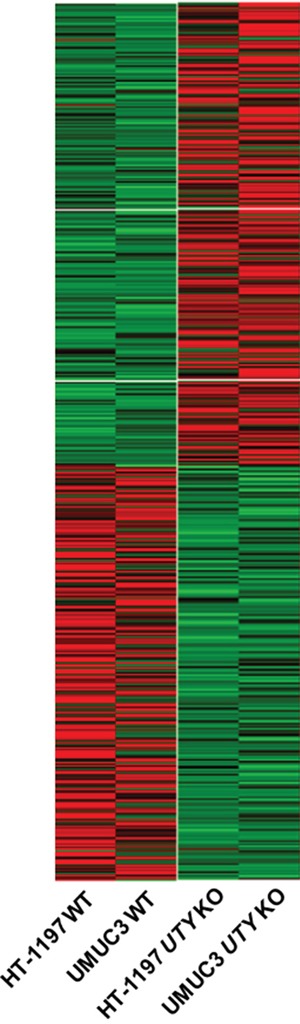
Changes in expression patterns in UBC cell lines after *UTY* knock-out Differentially expressed genes that exhibited the same trends (up/down-regulation) in both cell lines are shown in the heat map.

Genetic alterations in *UTY* have not been well-characterized. Therefore, we evaluated the effects of *UTY* knock-out on gene expression patterns using RNA sequencing data. We analyzed gene expression in *UTY* knock-out HT-1197 and UMUC3 cells compared to wild-type control cells. We found that 307 genes were simultaneously upregulated and 268 genes downregulated by at least two-fold in *UTY* knock-out cells (Figure 4). Gene ontology analysis indicated that the downregulated genes were involved in cellular processes such as cell adhesion (P = 5.83E-04), T-cell activation (P = 0.0038), and cell-cell signaling (P = 0.010) (Supplementary Table S5).

## DISCUSSION

In this study, we analyzed genomic alterations in 40 patients with UBC using target capture exome sequencing. A total of 16 genes involved in chromatin modifying processes were altered in approximately 75% of UBC patients. Gene expression is regulated by complex processes involving chromatin remodeling and histone post-translational modifications. There is increasing evidence that epigenetic regulatory mechanisms can act as tumor suppressors in various cancers [13]. The chromatin modifying genes that were mutated in this study included histone acetyltransferases (*EP300* and *CREBBP*), histone methyltransferases (*MLL2*, *MLL3*, *NSD1*, and *SETD2*), histone demethylases (*UTX*, *UTY*, *KDM5A*, and *KDM5B*), and ATPase-dependent chromatin-modifying complexes (*SMARCA2*, *SMARCA4*, *SMARCC2*, *ARID1A, ARID1B*, and *CHD6*). Although recent studies have suggested that the chromatin modifying machinery may have a central role in UBC development, the underlying mechanisms are still unknown [1–3].

*UTX* belongs to the histone lysine demethylase (KDM) family. The KDM6 family includes *UTX* (*KDM6A*), *JMJD3* (*KDM6B*), and *UTY* (*KDM6AL*) [14, 15]. These proteins contain a Jumonji C (JmjC) domain, which uses ferrous iron and α-ketoglutarate for histone demethylation [16]. Members of the KDM6 family have high sequence homology in the JmjC domain. Specifically, *UTX* and *UTY* have similar domain organizations, including an N-terminal tetratricopeptide repeat domain and a C-terminal JmjC domain [17]. *UTX* escapes X chromosome inactivation [18], and biallelic inactivation is necessary for pathogenesis in females. *UTY* shares 88% amino acid similarity with *UTX* [18]. However, *UTY* appears to lack demethylase activity. Thus, the allelic role of *UTY* as the male homologue of *UTX* is uncertain [15, 19]. Genetic analysis using a mouse model of *UTX* and *UTY* mutation has suggested that *UTY* can compensate for *UTX* loss during embryonic development [20]. In addition, Walport et al. demonstrated that *UTY* is a functional histone lysine demethylase. Because the demethylase activity of *UTY* was not observed at a global level, it is likely that it acts in a target-specific manner [17].

The role of *UTY* in cancer cell proliferation has not been elucidated. In our study, *UTY* copy number loss was observed in 22.8% of male UBC patients. Interestingly, loss of *UTY* was more common in male patients with *UTX* mutations (66.7%, 6/9) than in those with wild type *UTX* (7.7%, 2/26). These results are consistent with those of a previous study in which somatic mutations in *UTX* were investigated in multiple tumor types. The rates of *UTY* loss were 81% in *UTX* mutant and and 49% in *UTX* wild type cell lines derived from male UBC patients, respectively [7].

Recent data have suggested that only 3% of surviving ancestral genes on the Y chromosome play a critical role in gene expression. These genes also showed signs of dosage sensitivity [10]. While essential regulatory genes escape X chromosome inactivation in females, this group of genes on the Y chromosome is preserved in men thereby providing a male homologue for normal function. In our in vitro assays, *UTX*-*UTY* knock-outs in cell lines derived from male UBC patients indicated that loss of *UTY* led to cell proliferation in both *UTX* wild-type and *UTX* knock-out UBC cells. These findings suggest that *UTY* is also involved in the pathogenesis of UBC. We hypothesize that there are gene dosage effects. Although demethylase activity was not investigated in our study, loss of either *UTX* or *UTY* increased proliferation, indicating that *UTX* and *UTY* likely act as tumor suppressors in a dose-dependent manner. *UTY* may not fully compensate for the role of *UTX* to suppress tumor cell proliferation, but simultaneous loss of *UTX* and *UTY* might drive carcinogenesis. A two-hit model of *UTX*-*UTY* inactivation may be crucial for the development of UBC.

In summary, *UTX* was the most frequently mutated gene in UBC, and loss of *UTY* was frequently observed in patients with *UTX* mutations. Knock-out of both *UTX* and *UTY* increased UBC cell proliferation, suggesting that *UTY* acts as a tumor suppressor in UBC. While *UTX* escapes X chromosome inactivation in females, *UTY* may function as the male homologue of *UTX*, which could compensate for dosage imbalances and maintain the tumor suppressive effects in males. Simultaneous loss of *UTX* and *UTY* may be crucial for carcinogenesis. Therefore, the functional role of Y-linked genes and X-Y gene pairs should not be ignored.

## MATERIALS AND METHODS

### Study population and sample collection

We prospectively collected clinical information, tissue, and blood samples from patients undergoing therapy for UBC. This study was approved by the Institutional Review Board of Ewha Medical Center (IRB No.13-40-01), and all patients provided informed consent for tissue banking and genetic testing. Blood samples were collected prior to surgery for germline DNA extraction. Donated tumor tissue was stored as fresh frozen samples after surgery. The pathology was reviewed by a genitourinary pathologist (S.H. Park) and the tumor characteristics analyzed. We excluded patients who had undergone previous chemotherapy or radiotherapy and patients with upper urinary tract urothelial cancer. We performed genetic analysis on a total of 40 patients with UBC. Our samples consisted of 40 tumor tissues and 40 matched blood samples.

### Target gene selection

We selected 128 genes for target capture sequencing. Gene selection was based on recent large-scale studies and major pathways known to be involved in UBC. We performed hybridization to capture genomic regions of interest, which included 3,060 exons and 794,525 bases. A list of target genes that were sequenced is shown in Supplementary Table S2.

### Target capture and next-generation sequencing

DNA was extracted from blood and tissue samples using the DNeasy Blood and Tissue Kit (Qiagen, USA). For target capture, DNA from bladder cancer and matched blood samples was sheared into approximately 180 bp fragments, end-repaired, dA-tailed, and adapter-ligated using Illumina adapter pairs. Hybridization probes (Celemics, Korea) were mixed with the target DNA and then separated by bead purification. Target capture libraries were sequenced with the HiSeq 2500 platform (Illumina, USA) using 2 × 150 bp paired-end runs. The target sequencing depth and coverage are shown in Supplementary Table S3. Target sequencing data are accessible at the Sequence Read Archive (accession number [SRR3056095]).

### Somatic variant calling

The Novoalign software was used to map the sequence reads to the UCSC human genome (GRCh37/hg19). Local alignment and duplication removal were performed with the Genome Analysis Tool Kit (GATK) and Picard software. MuTect (version 1.0.287783) and VarScan (version 2.3.6) were used to call SNVs, and somatic indel calling was performed with the Indel Detector in GATK version 1.4-2. Mutation candidates at various loci were annotated with ANNOVAR. False positive indels were manually removed using the Integrated Genome Viewer (IGV version 1.8.0.).

### Detection of copy number alterations

We used the copy number detection mode in VarScan version 2.3.6. to compare differences in depth between paired tumor and blood samples. The copyCaller mode of VarScan along with the DNAcopy library included in R version 2.15.3 were used to classify copy number gain or loss. A log_2_ copy number ratio for a target > 1.0 was regarded as copy number gain and a ratio < -1.0 as copy number loss. Altered copy number regions were annotated to the target gene regions, and only regions that overlapped with the target region were included in the analysis.

### Sanger validation of randomly selected somatic mutations

For Sanger validation, candidate loci with allele frequencies between 0.1–0.9 were randomly selected to ensure the accuracy of the called loci. We selected a total of 47 loci (19.8% of the total mutations) including 43 SNVs and 4 indels. The primers used for Sanger sequencing are shown in Supplementary Table S5. PCR reactions consisted of 10 μL of KAPA HiFi HotStart ReadyMix PCR Mix (Kapa Biosystems, USA), 1 μL of each primer, and 8 μL of distilled water. The PCR conditions were as follows: 3 minutes at 95°C, 30 cycles of 30 seconds at 95°C, 30 seconds at 60°C, 30 seconds at 72°C, and 10 minutes at 72°C. PCR products were purified using the QIAquick Gel Extraction Kit (Qiagen, USA) and sent for Sanger sequencing (Macrogen, Korea). Sequencing data were analyzed with SeqMan (DNASTAR, USA).

### Validation of *UTY* copy number loss using TaqMan assays

We validated *UTY* gene copy number losses in eight bladder cancer patients using the TaqMan copy number validation method with a probe that targeted the region of interest (ChrY:15417296, Assay ID: Hs01026361_cn; Invitrogen, USA). We analyzed all 8 candidate samples with copy number losses in tumor-normal pairs. Additionally, one sample from a male patient with a normal *UTY* copy number and one sample from a female patient were assessed (Supplementary Figure S2). The mixture for real-time PCR consisted of 10 μL of 2X TaqMan^®^ Genotyping Master Mix (Applied Biosystems, USA), 1 μL of the TaqMan^®^ Copy Number Assay Reagent for the target of interest, 1 μL of the TaqMan^®^ Copy Number Reference Assay Reagent with RNase P (Invitrogen, USA), 4 μL of distilled water, and 4 μL of genomic DNA (5 ng/μL). PCR was performed according to the manufacturer's protocol. Copy number calls were based on real-time PCR data that was analyzed using the CopyCaller^®^ Software v2.0 (Applied Biosystems, USA).

### *UTX-* and *UTY*-targeted Cas9 lentivirus vector synthesis

To generate Cas9 knock-outs, we used the GeCKO gene knock-out protocol [12] and selected sgRNA sequences for *UTX* and *UTY* (Supplementary Figure S3). Oligo pairs (IDT, USA) for *UTX* and *UTY* were designed using the GeCKO lentiviral CRISPR toolbox protocol. We mixed 1 μL of each oligo pair (from 100 μM stocks) for each sgRNA with 8 μL of distilled water, 1 μL of T4 ligase buffer (Enzymatics, USA), and 0.5 μL of T4 PNK (Enzymatics, USA). Oligo pair annealing and phosphorylation were performed using the following conditions: 30 minutes at 37°C, 5 minutes at 95°C, 0.1°C per second to 25°C. After annealing, the oligo duplex was diluted with distilled water (1/200) and ligated to enzymatically digested lentiCRISPRv2 vector (Addgene, USA). We then incubated 2 μL of lentivirus plasmid (878 ng/μL), 2 μL of BsmBI (NEB, USA), and 6 μL of enzyme buffer (NEB, USA) at 37°C for 2 hours. The reaction product was purified using the QIAquick Gel Extraction Kit (Qiagen, USA) for ligation. The ligation reaction consisted of 1 μL of the diluted annealed oligo duplex, 2 μL of the digested lentivirus vector, 0.5 μL of ligase (Enzymetics, USA), 1 μL of 10X ligase buffer (Enzymetics, USA), and 7 μL of distilled water. The ligation reaction was then incubated for 30 minutes at room temperature. Finally, the ligated vector was transformed into Endura™ competent cells (Lucigen, USA), and colonies selected using colony PCR. Colony PCR products were validated with Sanger sequencing (Macrogen, Korea), and each accurately synthesized *UTX* and *UTY* target lentivirus vector was used in the virus production step.

### Production of *UTX*- and *UTY*-targeted lentiviruses

HEK293T cells (ATCC, USA) were cultured in Dulbecco's Modified Eagle's medium (DMEM) media (Life Technologies, USA) supplemented with 10% fetal bovine serum (Invitrogen, USA) and 1% penicillin/streptomycin (Life Technologies, USA). Six-well plates were coated with poly-D-lysine solution (Sigma-Aldrich, USA) at a concentration of 0.02 mg/mL before the HEK293T cells were seeded. Cells were seeded 1 day before transfection in a 6-well plate at 80% confluency, and the medium was changed to optiMEM (Life Technologies, USA) before transfection. For transfection, 1 μg of the target lentiviral vector, 0.25 μg of pMD2.G (Addgene, USA), and 0.75 μg of psPAX2 (Addgene, USA) were mixed with 5 μL of Lipofectamine 2000 (Life Technologies, USA) and 5 μl of the Plus Reagent (Life Technologies, USA). The medium was changed 16 hours after transfection. Virus supernatants were harvested 48 hours after transfection and again after 72 hours. Harvested supernatants were centrifuged (5 minutes, 1,500 rpm, 4°C) to pellet cells debris. The supernatants were collected using a syringe and filtered with a 0.45 μm filter.

### Lentiviral transduction

The HT-1197 and UMUC3 cell lines were used for all in vitro assays. Both cell types were cultured in RPMI 1640 (Life Technologies, USA) supplemented with 10% FBS (Seradigm, USA) and 1% penicillin/streptomycin (Life Technologies, USA). Cells were seeded 1 day prior to viral infection. Lentivirus infection was performed with a multiplicity of infection of 5. Each *UTX-* and *UT-* targeted lentivirus was combined with 8 μg polybrene (Sigma-Aldrich, USA) for several days. The medium was changed to RPMI 1640 24 hours post infection.

### Validation of target gene knock-out with surveyor assays and western blot analysis

To validate Cas9-mediated target site cleavage, we performed surveyor assays with the Surveyor^®^ Mutation Detection Kit (IDT, USA). The expression of each target gene was analyzed by western blotting. Cell lysates for protein extraction were prepared from cells cultured in 6-well plates. The extracted proteins were quantified using the Bradford protein assay (Bio-Rad, USA). Western blotting was performed according to standard protocols. The primary antibodies that were used for immunostaining were the following: UTX (Abcam, USA, product ID: ab91231; 1:1,000), UTY (Abcam, USA, product ID: ab91236; 1:1,000), GAPDH (Abcam, USA, product ID: ab8245; 1:1,000). The secondary rabbit-anti-mouse HRP antibody was also purchased from Abcam (product ID: ab6728; 1:1,000). Western blots were imaged using the Clarity™ Western ECL Substrate (Bio-Rad, USA). The results of the surveyor assays are shown in Supplementary Figure S3. The western blots are shown in Supplementary Figure S4.

### Proliferation assays

MTT assays (Sigma-Aldrich, USA) were performed at the indicated time-points using the manufacturer's protocol to evaluate the effects of Cas9-mediated target knock-down on proliferation. BrdU incorporation assays of cellular proliferation were performed using the BrdU Cell Proliferation Assay Kit (Cell Signaling Technology, USA) and the manufacturer's protocol (2 hour incubation with the BrdU detection solution). We also measured the total cell number at each time-point. On the day the measurements were performed, the cells were detached using trypsin and then counted using a LUNA™ Automated Cell Counter (Logos Biosystems, USA). Cell counting and MTT assays were performed every second day starting on the day of seeding. Every experiment was performed with 6-plex conditions.

### Gene expression analysis in *UTY* knock-out cells using RNA sequencing

RNA was extracted from *UTY* knock-out and wild type control cells (both the HT-1197 and UMUC3 cell lines). The RNeasy Mini Kit (Qiagen, USA) was used to extract total RNA from candidate cells according to the manufacturer's protocol. RNA sequencing libraries were prepared using the TruSeq Standard mRNA Library Prep Kit v2 (Illumina, USA). RNA libraries were sequenced on the Hiseq2500 platform (Illumina, USA) with 2 × 150 bp paired-end runs. RNA sequencing data were processed for differential gene expression analysis using the TopHat and Cufflinks protocols [21]. We selected gene sets that were upregulated or downregulated more than two-fold in both HT-1197 and UMUC3 knock-out cells (Supplementary Figure S5). Gene ontology analysis was performed using the DAVID web-based software (https://david.ncifcrf.gov/).

### Statistical analysis

We compared proliferation between cell lines using Mann-Whitney U tests. The Statistical Package for Social Science for Windows, version 18.0 (SPSS, USA) was used for statistical analyses. A P-value < 0.05 was considered significant and all P-values were two-sided.

## SUPPLEMENTARY FIGURES AND TABLES




