# Supplementary material for: Target sequencing and CRISPR/Cas editing reveal simultaneous loss of UTX and UTY in urothelial bladder cancer
Source: Oncotarget. 2016 Aug 11;7(39):63252–60. doi: 10.18632/oncotarget.11207 (PMC5325361; doi:10.18632/oncotarget.11207)
Supplement: Supplementary file 1 [file oncotarget-07-63252-s001.pdf]

Target sequencing and CRISPR/Cas editing reveal simultaneous loss of *UTX* and *UTY* in urothelial bladder cancer

SUPPLEMENTARY FIGURES AND TABLES

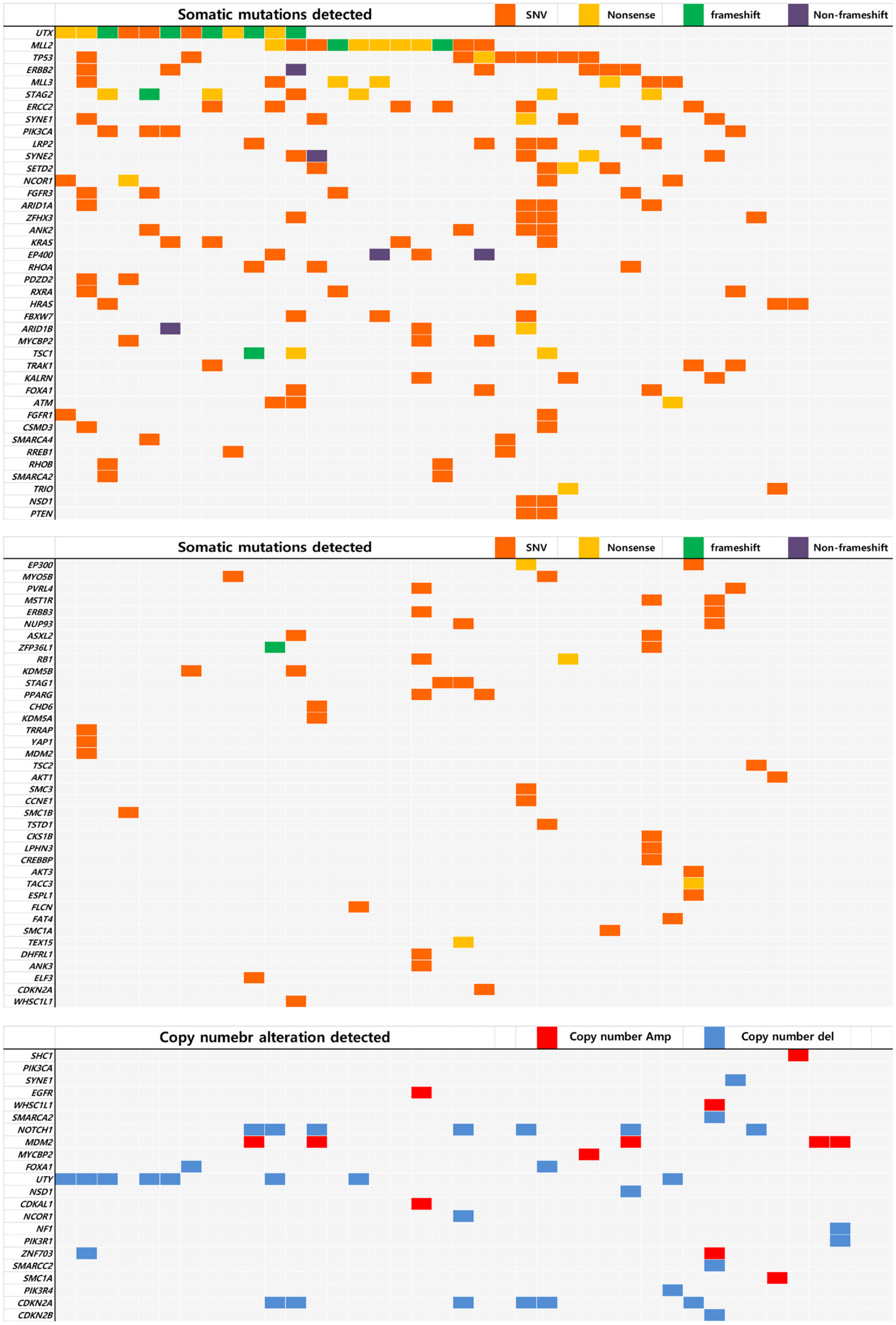

Supplementary Figure S1: Total somatic alteration profile of target capture sequencing.

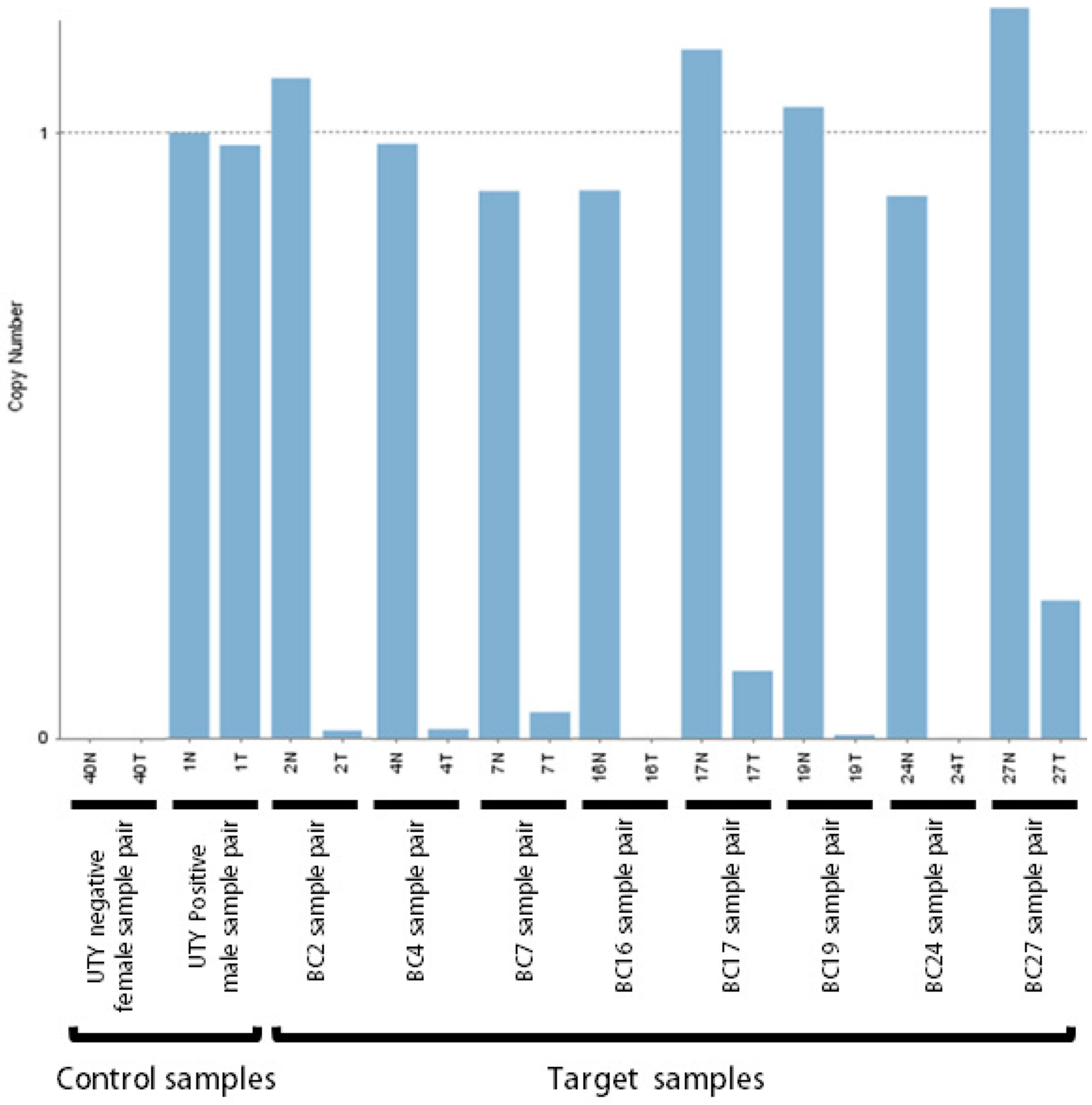

Supplementary Figure S2: TaqMan copy number validation of UTY from target and control samples. UTY positive male sample indicates male patient without UTY copy number loss detected among 35 sequenced male patients (T, tumor sample; N, blood sample). Copy number calls were based on real-time PCR data with CopyCaller® Software v2.0.

Gene	sgRNA sequence	chromosome	Target exon
UTX	TTGGATAATCTTCCAATAAG	X	3
UTY	GTCTGTTAGCCTGACAGTCG	Y	1

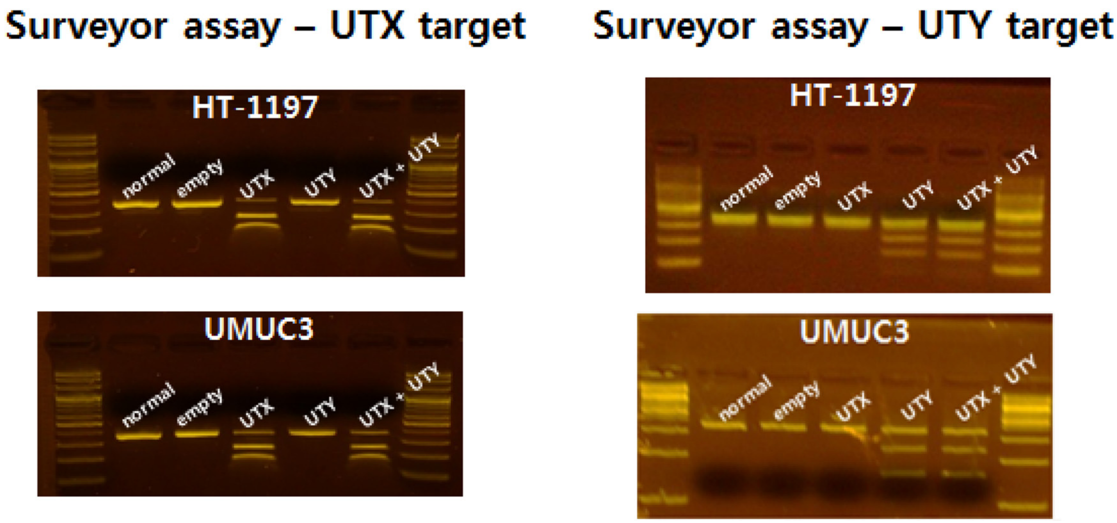

Supplementary Figure S3: Sequences used for target lentivirus synthesis and surveyor assay gel loading results.

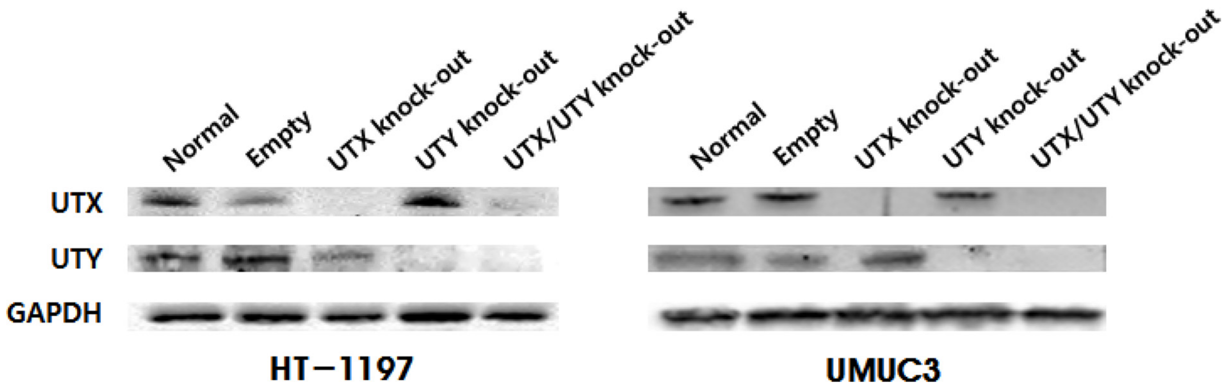

Supplementary Figure S4: Western blotting assay for validating target protein expression.

Up-regulated Genes	
**Term**	**Genes symbol**
GO:0007155~cell adhesion	IBSP, CLDN16, ICAM4, LGALS4, CDHR3, NINJ2, SIGLEC10, COL2A1, CLDN11, CTNNA3, GP5, PCDHB18, ITGB7, CD209, NPHS1, CD6
GO:0022610~biological adhesion	IBSP, CLDN16, ICAM4, LGALS4, CDHR3, NINJ2, SIGLEC10, COL2A1, CLDN11, CTNNA3, GP5, PCDHB18, ITGB7, CD209, NPHS1, CD6
GO:0006811~ion transport	CLDN16, PLCZ1, TF, TFR2, GLRA2, SCN2A, ATP1B4, ATP1A2, GRIA4, GJA4, KCNA7, ATP5EP2, ZACN, PLN, RACGAP1P, CAMK2B
GO:0016337~cell-cell adhesion	CLDN16, ICAM4, PCDHB18, CD209, CDHR3, NINJ2, COL2A1, CLDN11, CTNNA3
GO:0042110~T cell activation	ZBTB32, TNFSF14, TREML2, FOXP3, CD74, RHOH
GO:0046649~lymphocyte activation	ZBTB32, TNFSF14, TREML2, FOXP3, CD74, IL10, RHOH
GO:0001775~cell activation	PLCZ1, ZBTB32, TNFSF14, TREML2, FOXP3, CD74, IL10, RHOH
GO:0007267~cell-cell signaling	LEP, EFNB3, CRB1, SYN1, NEUROD1, ADRA1A, FGF12, ATP1A2, GRIA4, GJA4, IL10, WNT8B
GO:0046903~secretion	CLDN16, LEP, SYN1, TACR2, NPHS1, MS4A2, NEUROD1, ANXA2P3
GO:0002694~regulation of leukocyte activation	PLA2G10, TNFSF14, MS4A2, FOXP3, CD74, IL10
GO:0030001~metal ion transport	PLCZ1, TF, PLN, TFR2, SCN2A, ATP1B4, CAMK2B, ATP1A2, GJA4, KCNA7
GO:0050865~regulation of cell activation	PLA2G10, TNFSF14, MS4A2, FOXP3, CD74, IL10
GO:0045321~leukocyte activation	ZBTB32, TNFSF14, TREML2, FOXP3, CD74, IL10, RHOH
GO:0006790~sulfur metabolic process	GAL3ST3, GGT6, BGN, GGT3P, CHST5
GO:0015674~di-, tri-valent inorganic cation transport	PLCZ1, TF, PLN, TFR2, CAMK2B, GJA4
GO:0006812~cation transport	PLCZ1, TF, ATP5EP2, PLN, TFR2, SCN2A, ATP1B4, CAMK2B, ATP1A2, GJA4, KCNA7
GO:0051046~regulation of secretion	LEP, PLA2G10, MS4A2, NEUROD1, FOXP3, IL10
GO:0001880~Mullerian duct regression	AMHR2, SMAD9
GO:0051241~negative regulation of multicellular organismal process	PLN, ATP1A2, FOXP3, IL10, ANXA2P3
GO:0050868~negative regulation of T cell activation	FOXP3, CD74, IL10
GO:0046651~lymphocyte proliferation	ZBTB32, TNFSF14, IL10
GO:0006029~proteoglycan metabolic process	GAL3ST3, BGN, COL2A1

Down-regulated Genes	
**Term**	**Genes symbol**
GO:0003001~generation of a signal involved in cell-cell signaling	P2RX7, GRM2, NTRK2, PDX1, GAL
GO:0032989~cellular component morphogenesis	P2RX7, EPHA7, CD3G, XIRP1, PDPN, NKX2-8, POU4F3, CNTN2, NRXN1, DCLK1
GO:0007586~digestion	P2RX7, GALR2, NPC1L1, MEP1B, AMY1A
GO:0006812~cation transport	KCNJ5, P2RX7, TRPC4, SLC12A8, SCN3A, LCK, NOX1, KCNH6, ATP12A, KCNE4, ATP5L2, ATP13A4
GO:0045761~regulation of adenylate cyclase activity	SSTR5, ADCY1, GRM2, GALR2, NTRK2
GO:0031279~regulation of cyclase activity	SSTR5, ADCY1, GRM2, GALR2, NTRK2
GO:0030817~regulation of cAMP biosynthetic process	SSTR5, ADCY1, GRM2, GALR2, NTRK2
GO:0051339~regulation of lyase activity	SSTR5, ADCY1, GRM2, GALR2, NTRK2
GO:0030814~regulation of cAMP metabolic process	SSTR5, ADCY1, GRM2, GALR2, NTRK2
GO:0000902~cell morphogenesis	P2RX7, EPHA7, CD3G, PDPN, NKX2-8, POU4F3, CNTN2, NRXN1, DCLK1
GO:0048878~chemical homeostasis	SSTR5, P2RX7, XIRP1, LCK, NOX1, GALR2, FGF23, NPC1L1, POU3F2, PDX1, ATP12A
GO:0030030~cell projection organization	P2RX7, EPHA7, NKX2-8, GALR2, POU4F3, CNTN2, TEKT2, NRXN1, DCLK1
GO:0030808~regulation of nucleotide biosynthetic process	SSTR5, ADCY1, GRM2, GALR2, NTRK2
GO:0030802~regulation of cyclic nucleotide biosynthetic process	SSTR5, ADCY1, GRM2, GALR2, NTRK2
GO:0007166~cell surface receptor linked signal transduction	GPR83, WFIKKN2, ADCY1, CGB5, GPR68, ITGAM, IAPP, GALR2, QRFP, PTGER1, OR5P3, OR2AG2, CD3G, GPR133, CSNK1A1L, FGF23, GAL, PTGFR, FRZB, SSTR5, P2RX7, EPHA7, GRM2, LCK, NTRK2, RASD1
GO:0030799~regulation of cyclic nucleotide metabolic process	SSTR5, ADCY1, GRM2, GALR2, NTRK2
GO:0021869~forebrain ventricular zone progenitor cell division	POU3F3, POU3F2
GO:0006140~regulation of nucleotide metabolic process	SSTR5, ADCY1, GRM2, GALR2, NTRK2
GO:0015672~monovalent inorganic cation transport	KCNJ5, SLC12A8, SCN3A, NOX1, KCNH6, ATP12A, KCNE4, ATP5L2
GO:0006928~cell motion	EPHA7, NOX1, POU4F3, CNTN2, TEKT2, POU3F3, POU3F2, NRXN1, DCLK1, ITGAM
GO:0006954~inflammatory response	CIITA, P2RX7, PDPN, NOX1, C1R, GPR68, GAL, CD180
GO:0031175~neuron projection development	EPHA7, NKX2-8, GALR2, POU4F3, CNTN2, NRXN1, DCLK1
GO:0007409~axonogenesis	EPHA7, NKX2-8, POU4F3, CNTN2, NRXN1, DCLK1
GO:0048666~neuron development	EPHA7, NKX2-8, GALR2, NTRK2, POU4F3, CNTN2, NRXN1, DCLK1
GO:0048667~cell morphogenesis involved in neuron differentiation	EPHA7, NKX2-8, POU4F3, CNTN2, NRXN1, DCLK1
GO:0030182~neuron differentiation	EPHA7, NKX2-8, GALR2, NTRK2, POU4F3, CNTN2, POU3F2, NRXN1, DCLK1
GO:0048812~neuron projection morphogenesis	EPHA7, NKX2-8, POU4F3, CNTN2, NRXN1, DCLK1

Supplementary Figure S5: Total up/down-regulated gene list from RNA-seq analysis. Genes with fold-change >=2 are listed.

Supplementary Table S1: Clinical information of bladder cancer patient samples

See Supplementary File1

Supplementary Table S2: List of target sequenced genes

See Supplementary File1

Supplementary Table S3: Coverage of target sequencing samples. (T, tumor sample; N, blood sample)

See Supplementary File1

Supplementary Table S4: Total mutated loci information of target genes

See Supplementary File1

Supplementary Table S5: Sequenece of primers used for sanger validation

See Supplementary File1
