# Supplementary material for: Target sequencing and CRISPR/Cas editing reveal simultaneous loss of UTX and UTY in urothelial bladder cancer
Source: Oncotarget. 2016 Aug 11;7(39):63252–60. doi: 10.18632/oncotarget.11207 (PMC5325361; doi:10.18632/oncotarget.11207)
Supplement: Supplementary file 2 [file oncotarget-07-63252-s002.doc]

**Table S1.** Clinical information of bladder cancer patient samples

| **Case no** | **Sex** | **Age (years)** | **Smoking** | **Pack Years** | **Operation** | **T stage** | **N stage** | **Grade** | **Tumor contents (%)** |
| --- | --- | --- | --- | --- | --- | --- | --- | --- | --- |
| BC1 | Male | 78 | Never | 0 | RC | T2 | 0 | high | 80-90 |
| BC2 | Male | 68 | Never | 0 | TURB | Ta | Nx | low | 50-60 |
| BC3 | Male | 54 | Ever | 60 | TURB | T1 | Nx | high | 95 |
| BC4 | Male | 89 | Ever | 25 | TURB | T1 | Nx | high | 90 |
| BC5 | Male | 69 | Current | 141 | TURB | T1 | Nx | high | 30-40 |
| BC6 | Male | 60 | Never | 0 | TURB | Ta | Nx | low | 80-90 |
| BC7 | Male | 63 | Current | 48 | TURB | T1 | Nx | high | 90 |
| BC8 | Female | 77 | Never | 0 | TURB | Ta | Nx | low | 70-80 |
| BC9 | Male | 57 | Current | 60 | TURB | Ta | Nx | low | 80-90 |
| BC10 | Male | 58 | Ever | 10 | TURB | Ta | Nx | low | 80-90 |
| BC11 | Male | 77 | Ever | 50 | TURB | Ta | Nx | high | 80-90 |
| BC12 | Male | 72 | Current | 0 | TURB | Ta | Nx | low | 95 |
| BC13 | Male | 56 | Current | 20 | RC | T2 | 0 | high | 95 |
| BC14 | Male | 79 | Ever | 15 | TURB | T1 | Nx | high | 70-80 |
| BC15 | Male | 65 | Current | 40 | RC | T1 | 0 | high | 95 |
| BC16 | Male | 74 | Current | 50 | RC | T1 | 0 | low | 95 |
| BC17 | Male | 87 | Ever | 0 | TURB | T2 | Nx | high | 95 |
| BC18 | Male | 58 | Never | 0 | RC | T1 | 0 | low | 95 |
| BC19 | Male | 54 | Never | 0 | TURB | Ta | Nx | low | 95 |
| BC20 | Male | 58 | Ever | 90 | RC | T3 | N0 | high | 30 |
| BC21 | Male | 59 | Ever | 120 | RC | T3 | 0 | high | 30-40 |
| BC22 | Male | 50 | Current | 15 | RC | T3 | N1 | high | 95 |
| BC23 | Male | 83 | Never | 0 | RC | T4 | N1 | high | 80-90 |
| BC24 | Male | 52 | Ever | 31.5 | RC | Ta | 0 | low | 95 |
| BC25 | Female | 59 | Ever | 10.5 | RC | T2 | 0 | low | 50 |
| BC26 | Male | 71 | Ever | 44 | RC | T2 | 0 | high | 95 |
| BC27 | Male | 51 | Ever | 30 | RC | T2 | N2 | high | 50-60 |
| BC28 | Male | 82 | Ever | 50 | RC | T3 | N2 | high | 30-40 |
| BC29 | Male | 75 | Ever | 360 | RC | T4 | N2 | high | 90 |
| BC30 | Male | 59 | Current | 30 | TURB | Ta | Nx | high | 80-90 |
| BC31 | Female | 52 | Never | 0 | RC | T4 | N1 | high | 30-40 |
| BC32 | Male | 78 | Ever | 40 | TURB | Ta | Nx | low | 95 |
| BC33 | Male | 34 | Current | 10 | TURB | T1 | Nx | low | 95 |
| BC34 | Male | 71 | Ever | 25 | RC | T2 | N1 | high | 80-90 |
| BC35 | Male | 72 | Ever | 0 | TURB | Ta | Nx | low | 100 |
| BC36 | Male | 74 | Ever | 35 | RC | T3 | 0 | high | 30 |
| BC37 | Female | 91 | Current | 25 | TURB | T1 | Nx | high | 95 |
| BC38 | Male | 67 | Ever | 35 | RC | T3 | 0 | high | 70-80 |
| BC39 | Male | 70 | Ever | 16 | RC | T1 | 0 | high | 30 |
| BC40 | Female | 70 | Never | 0 | RC | T1 | Nx | high | 100 |

RC, Radical Cystectomy; TURB, Transurethral resection of bladder.

**Table S2.** List of target sequenced genes

| **No. of gene** | **Gene symbol** |  | **No. of gene** | **Gene symbol** |  | **No. of gene** | **Gene symbol** |  | **No. of gene** | **Gene symbol** |
| --- | --- | --- | --- | --- | --- | --- | --- | --- | --- | --- |
| #1 | *ARID1A* |  | #33 | *KIT* |  | #65 | *WHSC1L1* |  | #97 | *AKT1* |
| #2 | *MYCL* |  | #34 | *LPHN3* |  | #66 | *YWHAZ* |  | #98 | *MAPK8IP3* |
| #3 | *NRAS* |  | #35 | *ANK2* |  | #67 | *CSMD3* |  | #99 | *TSC2* |
| #4 | *TXNIP* |  | #36 | *FAT4* |  | #68 | *MYC* |  | #100 | *CREBBP* |
| #5 | *SHC1* |  | #37 | *INPP4B* |  | #69 | *SMARCA2* |  | #101 | *NUP93* |
| #6 | *MUC1* |  | #38 | *FBXW7* |  | #70 | *CDKN2A* |  | #102 | *ZFHX3* |
| #7 | *PVRL4* |  | #39 | *TRIO* |  | #71 | *CDKN2B* |  | #103 | *FANCA* |
| #8 | *ELF3* |  | #40 | *PDZD2* |  | #72 | *TSC1* |  | #104 | *TP53* |
| #9 | *KDM5B* |  | #41 | *NIPBL* |  | #73 | *RXRA* |  | #105 | *NCOR1* |
| #10 | *BTG2* |  | #42 | *PAIP1* |  | #74 | *NOTCH1* |  | #106 | *FLCN* |
| #11 | *AKT3* |  | #43 | *PIK3R1* |  | #75 | *ANK3* |  | #107 | *NF1* |
| #12 | *RHOB* |  | #44 | *DHFR* |  | #76 | *PTEN* |  | #108 | *KAT2A* |
| #13 | *ASXL2* |  | #45 | *NSD1* |  | #77 | *SMC3* |  | #109 | *BPTF* |
| #14 | *LRP2* |  | #46 | *FOXQ1* |  | #78 | *HRAS* |  | #110 | *MYO5B* |
| #15 | *PDK1* |  | #47 | *RREB1* |  | #79 | *CCND1* |  | #111 | *STK11* |
| #16 | *NFE2L2* |  | #48 | *E2F3* |  | #80 | *YAP1* |  | #112 | *DOT1L* |
| #17 | *ERBB4* |  | #49 | *CDKAL1* |  | #81 | *ATM* |  | #113 | *SMARCA4* |
| #18 | *TWIST2* |  | #50 | *CDKN1A* |  | #82 | *KMT2A* |  | #114 | *CCNE1* |
| #19 | *PPARG* |  | #51 | *CCND3* |  | #83 | *KDM5A* |  | #115 | *CEBPA* |
| #20 | *KAT2B* |  | #52 | *LAMA4* |  | #84 | *KRAS* |  | #116 | *AKT2* |
| #21 | *TRAK1* |  | #53 | *SYNE1* |  | #85 | *KMT2D* |  | #117 | *ERCC2* |
| #22 | *SETD2* |  | #54 | *ARID1B* |  | #86 | *ESPL1* |  | #118 | *BCL2L1* |
| #23 | *SMARCC1* |  | #55 | *TWIST1* |  | #87 | *ERBB3* |  | #119 | *E2F1* |
| #24 | *RHOA* |  | #56 | *EGFR* |  | #88 | *SMARCC2* |  | #120 | *CHD6* |
| #25 | *MST1R* |  | #57 | *TRRAP* |  | #89 | *MDM2* |  | #121 | *EP300* |
| #26 | *KALRN* |  | #58 | *KMT2E* |  | #90 | *EP400* |  | #122 | *SMC1B* |
| #27 | *PIK3R4* |  | #59 | *MET* |  | #91 | *RB1* |  | #123 | *UTX* |
| #28 | *STAG1* |  | #60 | *BRAF* |  | #92 | *KLF5* |  | #124 | *SMC1A* |
| #29 | *PIK3CA* |  | #61 | *RHEB* |  | #93 | *MYCBP2* |  | #125 | *AR* |
| #30 | *TACC3* |  | #62 | *KMT2C* |  | #94 | *FOXA1* |  | #126 | *STAG2* |
| #31 | *FGFR3* |  | #63 | *TEX15* |  | #95 | *SYNE2* |  | #127 | *SMARCA1* |
| #32 | *PDGFRA* |  | #64 | *ZNF703* |  | #96 | *ZFP36L1* |  | #128 | *UTY* |

**Table S3.** Coverage of target sequencing samples. (T, tumor sample; N, blood sample)

| **Sample** | | **Depth** | **1X (%)** | **10X (%)** | **25X (%)** | **50X (%)** | **100X (%)** |
| --- | --- | --- | --- | --- | --- | --- | --- |
| BC1 | 1T | 245.8904 | 99.729 | 98.5915 | 96.8176 | 93.0704 | 84.5455 |
|  | 1N | 565.3823 | 99.7368 | 99.0066 | 98.1411 | 96.2958 | 92.1151 |
| BC2 | 2T | 273.207 | 99.6471 | 98.3839 | 96.4279 | 93.0384 | 84.4355 |
|  | 2N | 521.775 | 99.7375 | 98.9998 | 97.9603 | 96.1954 | 91.663 |
| BC3 | 3T | 321.9258 | 99.7267 | 98.6634 | 96.9855 | 93.6373 | 86.2734 |
|  | 3N | 484.5759 | 99.6941 | 98.9355 | 97.7404 | 95.4818 | 90.1598 |
| BC4 | 4T | 381.0989 | 99.753 | 99.0156 | 97.6434 | 94.6828 | 88.1652 |
|  | 4N | 487.3225 | 99.7883 | 98.7884 | 97.5094 | 95.0052 | 89.4435 |
| BC5 | 5T | 626.022 | 99.8865 | 99.4857 | 98.6879 | 97.2084 | 93.9363 |
|  | 5N | 587.3808 | 99.7412 | 99.0694 | 98.1113 | 96.463 | 92.576 |
| BC6 | 6T | 219.0718 | 99.7563 | 98.7203 | 96.9974 | 92.9198 | 82.8086 |
|  | 6N | 586.0953 | 99.7635 | 99.0749 | 98.1897 | 96.3866 | 92.4815 |
| BC7 | 7T | 554.869 | 99.8405 | 99.3608 | 98.4229 | 96.3848 | 92.11 |
|  | 7N | 558.1095 | 99.7295 | 99.1198 | 98.2439 | 96.2804 | 92.0289 |
| BC8 | 8T | 574.5768 | 98.9452 | 98.2544 | 97.4118 | 95.7723 | 91.9625 |
|  | 8N | 523.9961 | 98.6429 | 97.9205 | 96.8143 | 94.8712 | 90.1337 |
| BC9 | 9T | 573.6133 | 99.8547 | 99.3175 | 98.5234 | 97.0421 | 93.1882 |
|  | 9N | 587.9348 | 99.7988 | 99.0337 | 98.2599 | 96.6408 | 92.6999 |
| BC10 | 10T | 427.1467 | 99.8257 | 99.1281 | 98.2491 | 96.6262 | 92.1789 |
|  | 10N | 528.6354 | 99.60359 | 98.86051 | 97.82829 | 95.83605 | 91.12228 |
| BC11 | 11T | 580.1355 | 99.8793 | 99.3618 | 98.5961 | 97.0163 | 93.3272 |
|  | 11N | 534.1454 | 99.6936 | 98.9971 | 97.9578 | 96.0229 | 91.3787 |
| BC12 | 12T | 469.2325 | 99.8492 | 99.3182 | 98.4024 | 96.75 | 92.3367 |
|  | 12N | 554.1184 | 99.7756 | 98.9156 | 97.8796 | 95.9321 | 91.5295 |
| BC13 | 13T | 529.3221 | 99.8409 | 99.2686 | 98.3425 | 96.776 | 92.7095 |
|  | 13N | 469.7563 | 99.7556 | 98.9122 | 97.6815 | 95.3611 | 89.6115 |
| BC14 | 14T | 221.801 | 99.7826 | 98.6554 | 96.7162 | 92.6608 | 82.2368 |
|  | 14N | 490.1946 | 99.7105 | 98.9127 | 97.688 | 95.4974 | 90.2139 |
| BC15 | 15T | 333.8093 | 99.7209 | 98.732 | 97.0797 | 93.262 | 84.6134 |
|  | 15N | 510.8284 | 99.7222 | 99.0725 | 98.1013 | 96.0825 | 91.3821 |
| **Sample** | | **Depth** | **1X (%)** | **10X (%)** | **25X (%)** | **50X (%)** | **100X (%)** |
| BC16 | 16T | 339.0939 | 99.6698 | 98.6837 | 96.8001 | 93.8983 | 87.5248 |
|  | 16N | 572.4344 | 99.7804 | 99.0089 | 98.0413 | 96.1779 | 92.053 |
| BC17 | 17T | 274.0708 | 99.6746 | 98.6972 | 96.9942 | 93.2388 | 85.0382 |
|  | 17N | 415.311 | 99.7187 | 98.8024 | 97.5893 | 95.4405 | 90.0588 |
| BC18 | 18T | 485.2958 | 99.8758 | 99.2385 | 98.2378 | 96.2378 | 91.6689 |
|  | 18N | 359.5708 | 99.5877 | 98.7409 | 97.3218 | 94.6217 | 88.2158 |
| BC19 | 19T | 376.3748 | 99.8124 | 98.7454 | 97.2279 | 94.0658 | 87.775 |
|  | 19N | 492.2835 | 99.8033 | 99.0267 | 97.8591 | 95.6341 | 90.4383 |
| BC20 | 20T | 426.8145 | 99.845 | 99.1158 | 98.0964 | 96.0213 | 90.515 |
|  | 20N | 445.5035 | 99.716 | 98.8898 | 97.5759 | 94.9681 | 88.9001 |
| BC21 | 21T | 480.1101 | 99.8147 | 99.2113 | 98.1976 | 96.0627 | 90.7544 |
|  | 21N | 531.4976 | 99.766 | 98.9983 | 97.9588 | 96.1008 | 91.5215 |
| BC22 | 22T | 422.3549 | 99.8374 | 99.0219 | 97.5904 | 95.2907 | 89.8059 |
|  | 22N | 484.9317 | 99.7748 | 98.9345 | 97.7933 | 95.3721 | 90.262 |
| BC23 | 23T | 487.2863 | 99.8151 | 99.0208 | 97.7109 | 94.9978 | 88.6752 |
|  | 23N | 479.6865 | 99.7525 | 98.9317 | 97.6682 | 95.3674 | 89.9051 |
| BC24 | 24T | 505.2407 | 99.7485 | 99.0153 | 97.6199 | 95.7644 | 91.3102 |
|  | 24N | 412.8963 | 99.7305 | 98.8263 | 97.3316 | 94.6512 | 88.2619 |
| BC25 | 25T | 373.5793 | 98.9547 | 97.9019 | 96.5907 | 93.7342 | 87.2256 |
|  | 25N | 581.7769 | 98.9931 | 97.9322 | 97.0178 | 95.322 | 91.2274 |
| BC26 | 26T | 310.6299 | 99.7853 | 98.86 | 97.366 | 94.5269 | 87.3309 |
|  | 26N | 525.4888 | 99.763 | 99.0126 | 98.1009 | 96.3394 | 92.2842 |
| BC27 | 27T | 601.6499 | 99.8147 | 99.447 | 98.5823 | 96.9231 | 93.2706 |
|  | 27N | 554.0195 | 99.719 | 99.076 | 97.9713 | 95.9299 | 91.4435 |
| BC28 | 28T | 557.5627 | 99.8281 | 99.3733 | 98.537 | 96.8742 | 93.1173 |
|  | 28N | 407.6593 | 99.7748 | 98.9307 | 97.9034 | 95.7904 | 90.6756 |
| BC29 | 29T | 655.2487 | 99.892 | 99.4442 | 98.808 | 97.7389 | 94.8707 |
|  | 29N | 568.174 | 99.7884 | 99.1261 | 98.2666 | 96.5845 | 92.2851 |
| BC30 | 30T | 250.8439 | 99.8013 | 98.9315 | 97.4211 | 94.4992 | 86.4196 |
|  | 30N | 530.0686 | 99.8723 | 99.214 | 98.3107 | 96.3796 | 92.0041 |
| BC31 | 31T | 479.6323 | 98.6396 | 98.0462 | 97.2742 | 95.6344 | 91.5424 |
|  | 31N | 564.3385 | 98.8117 | 97.9641 | 97.0867 | 95.4703 | 91.5856 |
| **Sample** | | **Depth** | **1X (%)** | **10X (%)** | **25X (%)** | **50X (%)** | **100X (%)** |
| BC32 | 32T | 266.0032 | 99.6746 | 98.9588 | 97.431 | 94.1921 | 85.9465 |
|  | 32N | 559.9109 | 99.778 | 98.9568 | 97.8669 | 95.9126 | 91.4102 |
| BC33 | 33T | 131.6803 | 99.8405 | 99.2626 | 98.067 | 94.645 | 76.3097 |
|  | 33N | 502.9901 | 99.845 | 99.1046 | 98.1915 | 96.5864 | 92.3919 |
| BC34 | 34T | 489.0055 | 99.8547 | 99.3002 | 98.4843 | 96.8735 | 92.7345 |
|  | 34N | 590.3493 | 99.8698 | 99.1203 | 98.1645 | 96.5471 | 92.7749 |
| BC35 | 35T | 543.2407 | 99.8612 | 99.3374 | 98.6085 | 97.1906 | 93.2199 |
|  | 35N | 516.4045 | 99.8602 | 99.0998 | 97.9956 | 96.075 | 91.3579 |
| BC36 | 36T | 387.5474 | 99.7578 | 99.1867 | 98.1387 | 96.2162 | 90.6016 |
|  | 36N | 547.8792 | 99.7663 | 99.1577 | 98.1807 | 96.3344 | 92.0971 |
| BC37 | 37T | 543.2641 | 98.7947 | 98.2066 | 97.3166 | 95.6464 | 91.5487 |
|  | 37N | 362.7074 | 98.4876 | 97.5888 | 96.3146 | 93.7793 | 87.9004 |
| BC38 | 38T | 570.8222 | 99.876 | 99.4232 | 98.6554 | 97.1841 | 93.8481 |
|  | 38N | 574.2985 | 99.8199 | 99.0998 | 98.1966 | 96.3484 | 92.2719 |
| BC39 | 39T | 271.7328 | 99.8194 | 98.9187 | 97.7675 | 95.0285 | 87.5179 |
|  | 39N | 532.8555 | 99.7395 | 99.0407 | 97.8329 | 95.7182 | 90.7933 |
| BC40 | 40T | 475.3835 | 98.7346 | 98.195 | 97.3008 | 95.6945 | 91.5345 |
|  | 40N | 530.9002 | 98.7183 | 97.9877 | 96.8957 | 95.0027 | 90.5133 |

**Table S4.** Total mutated loci information of target genes.

| **Gene** | **Mutation type** | **Chromosome** | **Position** | **Ref allele** | **Alt allele** | **Sample** |
| --- | --- | --- | --- | --- | --- | --- |
| *RHOA* | Missense | 3 | 49397742 | G | A | BC1 |
| *SYNE1* | Missense | 6 | 152510440 | G | A | BC1 |
| *CHD6* | Missense | 20 | 40045851 | G | C | BC1 |
| *SYNE2* | Non-Frameshift Indel | 14 | 64469634 | TTTT | T | BC1 |
| *UTX* | Nonsense | X | 44922760 | C | T | BC2 |
| *RHOA* | Missense | 3 | 49412884 | C | T | BC3 |
| *PIK3CA* | Missense | 3 | 178936082 | G | A | BC3 |
| *FGFR3* | Missense | 4 | 1806099 | A | G | BC3 |
| *ERBB2* | Missense | 17 | 37868208 | C | T | BC3 |
| *ARID1A* | Missense | 1 | 27087350 | C | G | BC4 |
| *ARID1A* | Nonsense | 1 | 27087961 | C | T | BC4 |
| *ARID1A* | Missense | 1 | 27101517 | C | T | BC4 |
| *PDZD2* | Missense | 5 | 32088230 | C | T | BC4 |
| *SYNE1* | Missense | 6 | 152763242 | G | A | BC4 |
| *TRRAP* | Missense | 7 | 98592295 | C | T | BC4 |
| *MLL3* | Missense | 7 | 151874395 | C | T | BC4 |
| *CSMD3* | Missense | 8 | 113668533 | G | T | BC4 |
| *RXRA* | Missense | 9 | 137328444 | C | T | BC4 |
| *YAP1* | Missense | 11 | 102100541 | G | A | BC4 |
| *MDM2* | Missense | 12 | 69233180 | G | A | BC4 |
| *TP53* | Missense | 17 | 7578406 | C | T | BC4 |
| *UTX* | Nonsense | X | 44913136 | C | T | BC4 |
| *TP53* | Missense | 17 | 7578513 | C | A | BC5 |
| *SMARCA4* | Missense | 19 | 11145753 | G | T | BC5 |
| *RHOB* | Missense | 2 | 20647450 | C | T | BC7 |
| *PIK3CA* | Missense | 3 | 178928234 | G | C | BC7 |
| *PIK3CA* | Missense | 3 | 178936091 | G | A | BC7 |
| *SMARCA2* | Missense | 9 | 2039864 | C | A | BC7 |
| *STAG2* | Nonsense | X | 123197896 | C | T | BC7 |
| *UTX* | Frameshift Indel | X | 44922728 | ACTCAT | A | BC7 |
| *TRIO* | Missense | 5 | 14389433 | G | C | BC8 |
| *HRAS* | Missense | 11 | 533874 | T | C | BC8 |
| *AKT1* | Missense | 14 | 105246482 | C | T | BC8 |
| *ARID1A* | Missense | 1 | 27101324 | G | A | BC10 |
| *LRP2* | Missense | 2 | 170063167 | G | A | BC10 |
| *ANK2* | Missense | 4 | 114279588 | G | A | BC10 |
| *FBXW7* | Missense | 4 | 153247243 | T | G | BC10 |
| *FBXW7* | Nonsense | 4 | 153268138 | G | A | BC10 |
| *PDZD2* | Nonsense | 5 | 32074726 | C | T | BC10 |
| *NSD1* | Missense | 5 | 176638136 | G | A | BC10 |
| *NSD1* | Missense | 5 | 176722404 | C | T | BC10 |
| *SYNE1* | Nonsense | 6 | 152671899 | G | A | BC10 |
| *ARID1B* | Nonsense | 6 | 157150547 | C | T | BC10 |
| *PTEN* | Missense | 10 | 89692829 | T | G | BC10 |
| *SMC3* | Missense | 10 | 112364037 | G | A | BC10 |
| *SYNE2* | Missense | 14 | 64473798 | G | A | BC10 |
| *SYNE2* | Missense | 14 | 64604559 | G | A | BC10 |
| *ZFHX3* | Missense | 16 | 72822357 | G | A | BC10 |
| *TP53* | Missense | 17 | 7577127 | C | T | BC10 |
| *CCNE1* | Missense | 19 | 30303620 | G | A | BC10 |
| *ERCC2* | Missense | 19 | 45860556 | G | A | BC10 |
| *EP300* | Nonsense | 22 | 41553245 | C | T | BC10 |
| *MYCBP2* | Missense | 13 | 77751957 | T | C | BC11 |
| *NCOR1* | Nonsense | 17 | 16046984 | G | T | BC11 |
| *SMC1B* | Missense | 22 | 45804738 | C | G | BC11 |
| *UTX* | Frameshift Indel | X | 44923042 | CA | C | BC11 |
| *FGFR3* | Missense | 4 | 1806593 | C | G | BC12 |
| *MLL3* | Nonsense | 7 | 151855950 | G | A | BC12 |
| *RXRA* | Missense | 9 | 137300905 | C | T | BC12 |
| *MLL2* | Frameshift Indel | 12 | 49424071 | GC | G | BC12 |
| *MLL2* | Frameshift Indel | 12 | 49438251 | TC | T | BC12 |
| *ARID1A* | Missense | 1 | 27105846 | G | C | BC13 |
| *TSTD1* | Missense | 1 | 161007571 | C | G | BC13 |
| *SETD2* | Missense | 3 | 47165333 | C | G | BC13 |
| *ANK2* | Missense | 4 | 114269462 | G | C | BC13 |
| *FGFR1* | Missense | 8 | 38277245 | C | G | BC13 |
| *TSC1* | Nonsense | 9 | 135802635 | G | A | BC13 |
| *PTEN* | Missense | 10 | 89717708 | C | G | BC13 |
| *KRAS* | Missense | 12 | 25398285 | C | G | BC13 |
| *TP53* | Missense | 17 | 7577085 | C | T | BC13 |
| *STAG2* | Nonsense | X | 123179055 | G | A | BC13 |
| *PVRL4* | Missense | 1 | 161044049 | G | C | BC14 |
| *TRAK1* | Missense | 3 | 42244168 | C | A | BC14 |
| *PIK3CA* | Missense | 3 | 178919148 | C | G | BC14 |
| *RXRA* | Missense | 9 | 137328351 | C | T | BC14 |
| **Gene** | **Mutation type** | **Chromosome** | **Position** | **Ref allele** | **Alt allele** | **Sample** |
| *MST1R* | Missense | 3 | 49940201 | G | A | BC15 |
| *KALRN* | Missense | 3 | 124379790 | G | C | BC15 |
| *SYNE1* | Missense | 6 | 152708275 | C | T | BC15 |
| *ERBB3* | Missense | 12 | 56481922 | G | A | BC15 |
| *ERBB3* | Missense | 12 | 56481923 | G | A | BC15 |
| *SYNE2* | Missense | 14 | 64656897 | G | T | BC15 |
| *NUP93* | Missense | 16 | 56873513 | T | A | BC15 |
| *PIK3CA* | Missense | 3 | 178952085 | A | G | BC16 |
| *FGFR3* | Missense | 4 | 1807889 | A | G | BC16 |
| *ANK2* | Missense | 4 | 114264234 | G | C | BC16 |
| *SMARCA4* | Missense | 19 | 11144147 | G | A | BC16 |
| *UTX* | Missense | X | 44949141 | C | G | BC16 |
| *STAG2* | Frameshift Indel | X | 123176475 | ATGAC | A | BC16 |
| *PIK3CA* | Missense | 3 | 178916957 | G | C | BC17 |
| *KRAS* | Missense | 12 | 25398284 | C | T | BC17 |
| *ERBB2* | Missense | 17 | 37871571 | C | G | BC17 |
| *ARID1B* | Non-Frameshift Indel | 6 | 157099425 | A | AGCA | BC17 |
| *UTX* | Frameshift Indel | X | 44929415 | AAC | A | BC17 |
| *ARID1A* | Missense | 1 | 27105745 | G | A | BC18 |
| *CKS1B* | Missense | 1 | 154950527 | G | C | BC18 |
| *ASXL2* | Missense | 2 | 25978935 | C | T | BC18 |
| *LRP2* | Missense | 2 | 170150673 | C | T | BC18 |
| *MST1R* | Missense | 3 | 49940529 | C | T | BC18 |
| *LPHN3* | Missense | 4 | 62758632 | G | A | BC18 |
| *MLL3* | Missense | 7 | 151836323 | G | A | BC18 |
| *FOXA1* | Missense | 14 | 38061339 | G | A | BC18 |
| *CREBBP* | Missense | 16 | 3820848 | G | C | BC18 |
| *STAG2* | Nonsense | X | 123171455 | C | T | BC18 |
| *ARID1A* | Frameshift Indel | 1 | 27106107 | G | GG | BC18 |
| *MLL3* | Missense | 7 | 151891626 | G | A | BC19 |
| *ATM* | Missense | 11 | 108204695 | G | C | BC19 |
| *ATM* | Missense | 11 | 108205709 | G | A | BC19 |
| *MLL2* | Nonsense | 12 | 49438595 | G | C | BC19 |
| *EP400* | Missense | 12 | 132546701 | C | T | BC19 |
| *UTX* | Nonsense | X | 44929487 | C | T | BC19 |
| *ZFP36L1* | Frameshift Indel | 14 | 69257141 | GC | G | BC19 |
| *SETD2* | Nonsense | 3 | 47164591 | G | C | BC20 |
| *KALRN* | Missense | 3 | 124157792 | G | A | BC20 |
| *TRIO* | Nonsense | 5 | 14481383 | C | G | BC20 |
| *SYNE1* | Missense | 6 | 152576794 | C | G | BC20 |
| *SYNE1* | Missense | 6 | 152651516 | C | G | BC20 |
| *RB1* | Nonsense | 13 | 48881462 | C | T | BC20 |
| *TP53* | Missense | 17 | 7577538 | C | T | BC20 |
| *AKT3* | Missense | 1 | 244006427 | C | G | BC22 |
| *TRAK1* | Missense | 3 | 42244146 | C | A | BC22 |
| *TACC3* | Nonsense | 4 | 1730121 | C | A | BC22 |
| *ERCC2* | Missense | 19 | 45855787 | C | A | BC22 |
| *EP300* | Missense | 22 | 41513217 | C | T | BC22 |
| *EP300* | Missense | 22 | 41513365 | C | T | BC22 |
| *SYNE2* | Nonsense | 14 | 64680945 | G | T | BC23 |
| *TP53* | Missense | 17 | 7577559 | G | A | BC23 |
| *ERBB2* | Missense | 17 | 37868208 | C | A | BC23 |
| *MLL2* | Nonsense | 12 | 49422905 | C | T | BC24 |
| *STAG2* | Nonsense | X | 123199756 | C | T | BC24 |
| *KDM5B* | Missense | 1 | 202710561 | C | T | BC25 |
| *TP53* | Missense | 17 | 7577099 | C | G | BC25 |
| *UTX* | Missense | X | 44921967 | C | G | BC25 |
| *KRAS* | Missense | 12 | 25380275 | T | G | BC26 |
| *ERCC2* | Missense | 19 | 45872386 | A | G | BC26 |
| *STAG2* | Nonsense | X | 123199766 | C | G | BC26 |
| *UTX* | Frameshift Indel | X | 44969444 | G | GAT | BC26 |
| *FAT4* | Missense | 4 | 126372946 | C | T | BC27 |
| *MLL3* | Missense | 7 | 151836846 | C | G | BC27 |
| *ATM* | Nonsense | 11 | 108098418 | C | T | BC27 |
| *ATM* | Missense | 11 | 108199963 | C | A | BC27 |
| *NCOR1* | Missense | 17 | 15952267 | G | A | BC27 |
| *FBXW7* | Missense | 4 | 153247168 | T | C | BC29 |
| *MLL3* | Nonsense | 7 | 151859398 | G | C | BC29 |
| *MLL2* | Nonsense | 12 | 49432741 | G | A | BC29 |
| *EP400* | Non-Frameshift Indel | 12 | 132547137 | A | AGCA | BC29 |
| *RREB1* | Missense | 6 | 7246986 | G | A | BC30 |
| *MYO5B* | Missense | 18 | 47367806 | C | G | BC30 |
| *UTX* | Nonsense | X | 44922802 | C | T | BC30 |
| *SETD2* | Missense | 3 | 47165554 | G | C | BC32 |
| *MLL3* | Nonsense | 7 | 151921210 | C | T | BC32 |
| *ERBB2* | Missense | 17 | 37879854 | G | A | BC32 |
| *SMC1A* | Missense | X | 53409447 | A | G | BC32 |
| *STAG1* | Missense | 3 | 136219120 | C | T | BC34 |
| *ANK2* | Missense | 4 | 114176933 | G | A | BC34 |
| **Gene** | **Mutation type** | **Chromosome** | **Position** | **Ref allele** | **Alt allele** | **Sample** |
| *TEX15* | Nonsense | 8 | 30700956 | C | A | BC34 |
| *MLL2* | Missense | 12 | 49444222 | G | A | BC34 |
| *MLL2* | Nonsense | 12 | 49444286 | G | A | BC34 |
| *NUP93* | Missense | 16 | 56782199 | G | A | BC34 |
| *TP53* | Missense | 17 | 7576870 | C | T | BC34 |
| *TP53* | Missense | 17 | 7577127 | C | G | BC34 |
| *KRAS* | Missense | 12 | 25362830 | A | G | BC35 |
| *MLL2* | Nonsense | 12 | 49425638 | G | A | BC35 |
| *ERCC2* | Missense | 19 | 45873425 | T | C | BC35 |
| *PVRL4* | Missense | 1 | 161044536 | G | T | BC36 |
| *DHFRL1* | Missense | 3 | 93779907 | G | A | BC36 |
| *KALRN* | Missense | 3 | 124175535 | G | A | BC36 |
| *ARID1B* | Missense | 6 | 157522047 | C | A | BC36 |
| *ANK3* | Missense | 10 | 61833378 | C | G | BC36 |
| *MLL2* | Nonsense | 12 | 49427027 | G | A | BC36 |
| *MLL2* | Missense | 12 | 49445971 | C | T | BC36 |
| *ERBB3* | Missense | 12 | 56482537 | G | A | BC36 |
| *EP400* | Missense | 12 | 132502758 | G | A | BC36 |
| *EP400* | Nonsense | 12 | 132535163 | C | T | BC36 |
| *RB1* | Missense | 13 | 49037957 | C | T | BC36 |
| *MYCBP2* | Missense | 13 | 77831929 | G | C | BC36 |
| *ELF3* | Missense | 1 | 201983043 | C | G | BC37 |
| *LRP2* | Missense | 2 | 170044681 | G | A | BC37 |
| *RHOA* | Missense | 3 | 49397710 | C | T | BC37 |
| *TSC1* | Frameshift Indel | 9 | 135786019 | C | CA | BC37 |
| *UTX* | Frameshift Indel | X | 44894201 | GTAATCCC | G | BC37 |
| *LRP2* | Missense | 2 | 170136000 | G | C | BC38 |
| *PPARG* | Missense | 3 | 12434148 | C | G | BC38 |
| *CDKN2A* | Missense | 9 | 21974741 | C | G | BC38 |
| *MLL2* | Missense | 12 | 49433575 | C | T | BC38 |
| *MLL2* | Missense | 12 | 49434538 | C | G | BC38 |
| *MLL2* | Missense | 12 | 49434595 | C | G | BC38 |
| *MYCBP2* | Missense | 13 | 77831908 | G | C | BC38 |
| *FOXA1* | Missense | 14 | 38064167 | G | A | BC38 |
| *TP53* | Nonsense | 17 | 7578188 | C | A | BC38 |
| *TP53* | Missense | 17 | 7578257 | C | T | BC38 |
| *ERBB2* | Missense | 17 | 37881132 | G | A | BC38 |
| *EP400* | Non-Frameshift Indel | 12 | 132547093 | A | ACAG | BC38 |
| *RHOB* | Missense | 2 | 20647450 | C | G | BC39 |
| *STAG1* | Missense | 3 | 136221536 | C | G | BC39 |
| *SMARCA2* | Missense | 9 | 2060967 | A | G | BC39 |
| *ERCC2* | Missense | 19 | 45868168 | G | C | BC39 |
| *MLL2* | Frameshift Indel | 12 | 49446149 | TG | T | BC39 |
| *KDM5B* | Missense | 1 | 202701006 | G | A | BC40 |
| *ASXL2* | Missense | 2 | 26022340 | G | A | BC40 |
| *FBXW7* | Missense | 4 | 153247246 | T | C | BC40 |
| *WHSC1L1* | Missense | 8 | 38205323 | C | T | BC40 |
| *TSC1* | Nonsense | 9 | 135781467 | G | A | BC40 |
| *ATM* | Missense | 11 | 108138018 | G | A | BC40 |
| *MLL2* | Missense | 12 | 49424151 | C | A | BC40 |
| *MLL2* | Missense | 12 | 49431339 | G | A | BC40 |
| *FOXA1* | Missense | 14 | 38061468 | G | A | BC40 |
| *SYNE2* | Missense | 14 | 64690033 | G | C | BC40 |
| *ZFHX3* | Missense | 16 | 72830108 | C | G | BC40 |
| *STAG2* | Missense | X | 123197851 | G | A | BC40 |
| *ERBB2* | Non-Frameshift Indel | 17 | 37866348 | C | CTGTCTGTGCCGG | BC40 |
| *UTX* | Frameshift Indel | X | 44922822 | CTG | C | BC40 |

**Table S5.** . Sequenece of primers used for sanger validation

|  | **Sanger fwd primer** | **Sanger rev primer** | **chromosome** | **position** | **ref** | **alt** | **Detected allele freq** | **Mutation type** |
| --- | --- | --- | --- | --- | --- | --- | --- | --- |
| #1 | GAAGCAAATGTTCCAGTGTATAGG | GGAACAGTCCTATTGGATATAATCC | X | 44949141 | C | G | 0.909091 | SNV |
| #2 | TTGACAGATGAGACCAACAGG | GCAGAAAAGGGTCCATTGG | X | 44922760 | C | T | 0.899083 | SNV |
| #3 | AATGAAGAGTTCACAATCTTCAAC | GACTATCACCAAGACTTAACACTTG | X | 123197896 | C | T | 0.880952 | SNV |
| #4 | GTGGATGTCCTCAAAAGACTTG | CCTGTCCTCCTGCAGGATTC | 11 | 533874 | T | A | 0.8084 | SNV |
| #5 | ACACAACCAGCATTTACTTTTCC | ACGGACTCCAGGCTGAGAG | X | 44922728 | ACTCAT | A | 0.787313433 | indel |
| #6 | CAGAGGGATAGCAGACGAG | CATATATGTTCTGCCCTTGTCTC | 9 | 135786019 | C | CA | 0.742268041 | indel |
| #7 | GGAATTCCTAATGGGCCAAC | CTTCCCAGCGTTCTTGATGTG | X | 44922802 | C | T | 0.719512 | SNV |
| #8 | TAGACGATGACCGACCCCAC | GACAGGAGACAGGTGTTGC | 8 | 38277245 | C | G | 0.667961 | SNV |
| #9 | GCATTCAGGTTCTGAAACTTTC | TCCTCAGTGTTCGCCACTC | 12 | 49444222 | G | A | 0.63 | SNV |
| #10 | CTGCTGTAGTCGGTGGTAGA | GTGCTAACAACATCTCTTTAGGTGG | X | 123171455 | C | T | 0.603352 | SNV |
| #11 | GGAAGCATGTTCTAAAACTTACC | GTAGAGGGGCTCAACTGAC | X | 123197851 | G | A | 0.565891 | SNV |
| #12 | CCTCTATTGTTGGATCATATTCG | CCTTATGTGTGACATGTTCTAATATAG | 12 | 25398285 | C | G | 0.558912 | SNV |
| #13 | CATCGGAATTTGAAAACAAGC | GACCAAGTCACTCTTTCTATGC | 11 | 108199963 | C | A | 0.545455 | SNV |
| #14 | GTGGATGTCCTCAAAAGACTTGG | TGTCCTCCTGCAGGATTCC | 11 | 533874 | T | C | 0.507143 | SNV |
| #15 | GTTGTTTAAGGAAAATGCTAAGC | TTACTCTGCTCTCCTTGGC | 1 | 27087350 | C | G | 0.492683 | SNV |
| #16 | CACACCATATATTAACTTCTGAC | CAAAAATAAGTCAAGATAAACATGCA | X | 123176475 | ATGAC | A | 0.492424242 | indel |
| #17 | GTCTGGAGCAGGCAACCAGA | GCTTCATGGTCAAACAGCTCTCC | 1 | 27087961 | C | T | 0.468085 | SNV |
| #18 | GGAAGTAATGGAAACGTGC | CTCTTCTTCTCTCAAAGTGT | X | 44923042 | CA | C | 0.463476071 | indel |
| #19 | GTCCTTTAAAGTGACGTCC | AGGTAGATCATCTCTTGCATAC | 6 | 152671899 | G | A | 0.4625 | SNV |
| #20 | CATCCAGAGTAGCACATACC | GGATAGCGATTCTCCTGACAGC | 12 | 49422905 | C | T | 0.458333 | SNV |
| #21 | CGGTAATGCTGCTCATGGTG | CTTGCTGCACTTCTCACACC | 17 | 37868208 | C | T | 0.457627 | SNV |
| #22 | TGACAGTGGAGCCTGTGTAC | CCACAATTTTGTCTAAAATCTTGG | 12 | 56481922 | G | A | 0.435484 | SNV |
| #23 | CTGGAACATCAAAGCCATCAAAAC | GACCAGGACTCCGGTAAGAG | 5 | 176638136 | G | A | 0.412935 | SNV |
| #24 | GATGACATTGCATACATTCGAAAG | CAGAGTGAGCTTTCATTTTCTCAG | 3 | 178952085 | A | G | 0.405063 | SNV |
| #25 | ATGGCAGTCAAACCTTCTCTC | ATCCTGCTTCTCGGGATAC | 3 | 178928234 | G | C | 0.396341 | SNV |
| #26 | TATGCATATTAAAACAAGATTTACCTC | ACCTTATGTGTGACATGTTC | 12 | 25398284 | C | T | 0.383929 | SNV |
| #27 | GTGCAGCGTCCAGTAGGAG | GCAAGATGCTCACGCTGAG | 14 | 38061339 | G | A | 0.379085 | SNV |
| #28 | GTGTTGAATCAATGGTTAACAG | GTATTTTGGCACTGTTGATTCAC | 14 | 64473798 | G | A | 0.375 | SNV |
| #29 | CCTGAGAACTGGAGGCTGC | ATACCGACTGCCATTTCTTTTG | 9 | 135781467 | G | A | 0.375 | SNV |
| #30 | CTTAGAACTATCTGGAATTTCTTCATC | GATGTAGCAGTTAGATCTCTG | 3 | 47165333 | C | G | 0.364532 | SNV |
| #31 | CTAGATTATGATGTGTTCCATG | GAATAAATGAGATCAAATGAATTACC | 13 | 49037957 | C | T | 0.352941 | SNV |
| #32 | GAGTGCTGTGTGAAATATTTG | AGGCTGTCACAGAAGTGAC | 12 | 132546701 | C | T | 0.348485 | SNV |
| #33 | CAGTGTGCAGGGTGGCAAG | AGGTCTCCCCAAGGCGCAC | 17 | 7577559 | G | A | 0.333333 | SNV |
| #34 | CGTTTAGCTTACCTTTTGCAGC | TACTTAAAGAGCAATACTCGC | 9 | 2060967 | A | G | 0.325 | SNV |
| #35 | CAATTTGATTTATAAGTATATGCCAA | CCTTACCAATACTCCATCCAC | 13 | 48881462 | C | T | 0.295082 | SNV |
| #36 | TAGAAGGTATATAAAATTACTGCTAC | GCATTATTTCCATATATAGTGTTAATTC | 8 | 113668533 | G | T | 0.282686 | SNV |
| #37 | CCATCACATTGCAACAGATG | TCAACACATGACTCTCTGG | 12 | 69233180 | G | A | 0.259259 | SNV |
| #38 | GCTCCTTTCACTCTCCACATC | CAACAGCTGCCTGAATGCAAAG | 3 | 47125385 | G | A | 0.2534 | SNV |
| #39 | TGAGAACAATATAGAGCAGT | GCATCTTGTACCATGCCAAG | 22 | 41513217 | C | T | 0.236641 | SNV |
| #40 | AGTCTGAGCACTGCTGGAG | CAAATATCTCCACCGTCCGAGAC | 4 | 126372946 | C | T | 0.2175 | SNV |
| #41 | GAAGACGACACAGGAGTTGATG | CAGCCAGCTACCTTGCCCTC | 16 | 3820848 | G | C | 0.201613 | SNV |
| #42 | ACCTTCCCAAAGAAAGAGAAG | GCTCCCTTACTTTAGATGATATG | 1 | 202710561 | C | T | 0.159722 | SNV |
| #43 | GTGATTGCATCTAATGTTTTCC | CACAGAGTTTTAGTTGTTCAG | 3 | 178919148 | C | G | 0.147059 | SNV |
| #44 | GTTTAAGTGAAAAGCCTTCA | CAATTGGAGCTGTTCCTTCC | 3 | 136219120 | C | T | 0.137615 | SNV |
| #45 | GCTCCAGGGTGTCAACTTAC | CTACCTGTCCCATGGAGCC | 12 | 49433575 | C | T | 0.128866 | SNV |
| #46 | CTTCCTTTGTCTGCCTTTACG | CAGAGTTTGTGTGCCCAAGG | 19 | 45872386 | A | G | 0.11976 | SNV |
| #47 | ATACACACACAGGCAGGAAC | TTGTGTTTCCCAGAGTGGAG | 6 | 152576794 | C | G | 0.105727 | SNV |
